# A real-world retrospective study of vitiligo associated with immune checkpoint inhibitors based on FAERS and TCGA databases

**DOI:** 10.1097/MD.0000000000049307

**Published:** 2026-06-12

**Authors:** Jingchang Ma, Zhibo Cai, Ya Luo, Xiaojun Li, Jinyi Li, Mingjun Hou, Dingheng Zhu

**Affiliations:** a Department of Dermatology, Air Force Hospital of Southern Theatre Command, Guangzhou, China; b Department of Dermatology, Dermatology Hospital, Southern Medical University, Guangzhou, China.

**Keywords:** FAERS, immune checkpoint inhibitors, pharmacovigilance, vitiligo

## Abstract

Immune checkpoint inhibitors (ICIs) have significantly improved outcomes in advanced malignancies but are frequently associated with immune-related adverse events. Vitiligo, a distinctive cutaneous immune-related adverse event, correlates with favorable prognosis in melanoma patients; however, its association with different ICI regimens and occurrence in nonmelanoma cancers remains inadequately characterized. We retrieved ICI-related vitiligo cases from the FDA Adverse Event Reporting System database between January 2015 and September 2025. Disproportionality analysis was performed using reporting odds ratio, proportional reporting ratio, Bayesian confidence propagation neural network, and multi-item gamma Poisson shrinker algorithms. The potential biological mechanisms underlying vitiligo induced by ICIs were examined using The Cancer Genome Atlas data. A total of 591 ICI-induced vitiligo cases were identified, wherein 358 (60.6%) and 177 (29.9%) were reported as anti-programmed cell death protein 1 and combination therapy. Combination therapy exhibited the highest crude incidence rate (0.57%), while anti-programmed death-ligand 1 monotherapy showed the lowest (0.06%). The ipilimumab–pembrolizumab combination demonstrated the highest reporting odds ratio (97.2, 95% CI: 58.1–162.4). Median time-to-onset varied significantly across ICI classes: anti-cytotoxic T-lymphocyte-associated protein 4 monotherapy (33 days), anti-programmed death-ligand 1 (50 days), combination therapy (61 days), and anti-programmed cell death protein 1 (72 days). Patients aged 65 to 79 years showed significantly delayed onset. Geographic analysis revealed prolonged time-to-onset in Asian patients versus Americans. Pan-cancer transcriptomic analysis demonstrated correlations between ICI-induced vitiligo and melanocyte development, innate immunity, and mitochondrial function. ICI-induced vitiligo exhibits significant heterogeneity in risk across treatment strategies, with combination therapy and anti-cytotoxic T-lymphocyte-associated protein 4 strategies conferring higher relative risks. Onset timing is substantially influenced by age and geographic factors. Integration of pharmacovigilance and transcriptomic data implicates coordinated effects of melanocyte-lineage antigens, innate immune and mitochondrial function pathways in vitiligo pathogenesis. This study aimed to systematically characterize the risk profile, onset timing, and potential biological mechanisms of ICI-induced vitiligo across different treatment strategies and cancer types.

## 1. Introduction

Immune checkpoint inhibitors (ICIs), encompassing monoclonal antibodies targeting programmed cell death protein 1 (PD-1), its ligand (PD-L1), and cytotoxic T-lymphocyte-associated protein 4 (CTLA-4), have fundamentally transformed the therapeutic landscape for advanced malignancies.^[1,2]^ By reinvigorating antitumor T-cell responses, these agents achieve durable clinical benefit in a subset of patients across multiple cancer types. However, the augmented immune activation frequently leads to a spectrum of immune-related adverse events (irAEs), which can involve any organ system and occasionally result in severe or life-threatening complications.^[3,4]^

Dermatologic toxicities represent among the most common irAEs, affecting 30% to 50% of treated patients, with manifestations ranging from maculopapular rash and pruritus to severe bullous disorders and toxic epidermal necrolysis.^[5,6]^ Vitiligo, characterized by acquired, progressive depigmentation resulting from autoimmune-mediated melanocyte destruction, has emerged as a distinctive cutaneous irAE with particular clinical significance.^[7,8]^ Unlike many other irAEs that necessitate treatment discontinuation, vitiligo development in melanoma patients has been consistently associated with improved objective response rates and prolonged survival, suggesting a mechanistic link between antimelanocyte immunity and antitumor efficacy.^[9,10]^ Nevertheless, the occurrence of ICI-induced vitiligo in nonmelanoma cancers and its relationship with specific ICI regimens remain insufficiently characterized.

The pathogenesis of ICI-induced vitiligo is thought to involve cross-reactivity between melanocyte differentiation antigens and tumor-associated antigens, leading to autoimmune-mediated melanocyte destruction.^[7]^ Despite increasing recognition of vitiligo as a clinically relevant irAE, large-scale pharmacoepidemiologic studies systematically evaluating its association with various ICI treatment strategies are scarce. Most existing evidence is derived from clinical trials, case series, or small cohort studies, which are limited by sample size, selection bias, and underreporting of irAEs. Furthermore, the temporal dynamics of vitiligo onset following ICI initiation and the potential biological pathways linking ICIs to vitiligo across diverse tumor types remain poorly understood.

Therefore, the primary aim of this study was to systematically evaluate the pharmacovigilance profile and potential biological mechanisms of ICI-induced vitiligo. Specifically, we aimed to: characterize the risk of vitiligo associated with different ICI treatment strategies using a large real-world adverse event database; investigate the time-to-onset (TTO) patterns of vitiligo following ICI initiation and identify factors influencing onset timing; and explore the transcriptomic signatures linked to ICI-induced vitiligo across different cancer types. We hypothesized that: combination ICI therapy would be associated with higher disproportionality signals for vitiligo compared to monotherapy; the TTO of vitiligo would vary significantly across ICI classes and be influenced by demographic and geographic factors; and ICI-induced vitiligo may involve transcriptomic pathways related to melanocyte function and immune regulation. In this study, we retrieved ICI-induced vitiligo data between 2015 and 2025 from the FDA adverse event reporting system (FAERS) database, performed disproportionality analysis, and explored the potential biological mechanisms associated with ICI-induced vitiligo in combination with The Cancer Genome Atlas (TCGA) data.

## 2. Methods

### 2.1. Data source

Pharmacovigilance data were obtained from the FAERS database, a spontaneous reporting system maintained by the U.S. Food and Drug Administration that aggregates adverse event reports submitted by healthcare professionals, consumers, and manufacturers.^[11]^ FAERS captures diverse clinical information, including patient demographics, drug exposures, indications, adverse event outcomes, and temporal relationships. For this analysis, we retrieved all adverse reaction reports from January 2015 to September 2025. Adverse events were coded using preferred terms from the Medical Dictionary for Regulatory Activities (MedDRA, version 25.0).

Transcriptomic data for pan-cancer pathway analysis were obtained from TCGA, a collaborative initiative of the National Cancer Institute and National Human Genome Research Institute that has generated comprehensive genomic, epigenomic, transcriptomic, and proteomic profiles of over 11,000 tumors across 33 cancer types. Specifically, we utilized precomputed single-sample gene set enrichment analysis scores for the PARADIGM pathway collection available through the UCSC Xena platform.^[12]^

### 2.2. Data processing procedure

**Figure 1.**
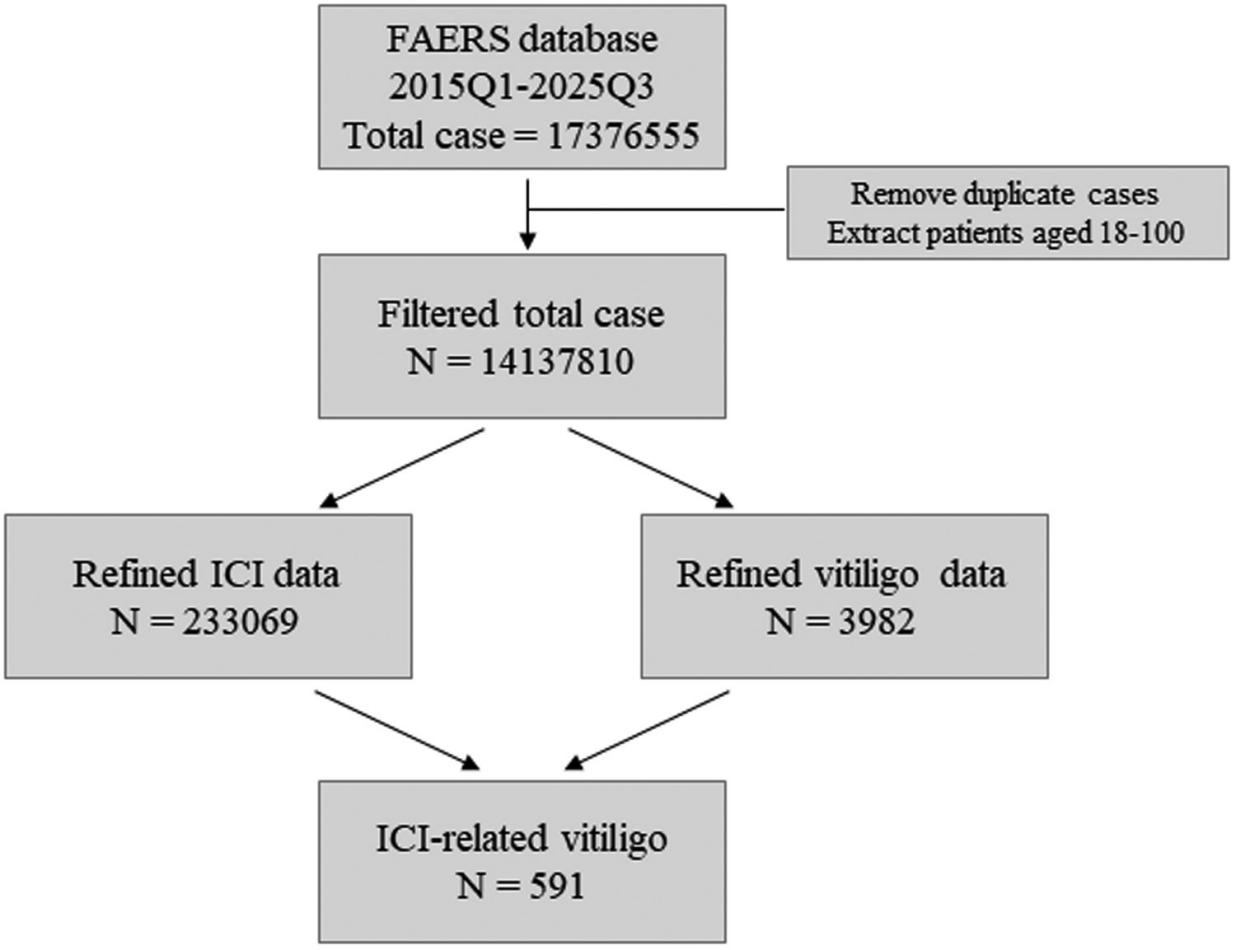
Data filtering process of FAERS performed in this study. FAERS = FDA adverse event reporting system, ICI = immune checkpoint inhibitor.

We implemented a systematic screening procedure for FAERS data (Fig. 1). Duplicate reports were excluded based on concordance of PRIMARYID, CASEID, and FDA_DT fields. We restricted analyses to patients aged 18 to 100 years to ensure adult populations and exclude potential pediatric reporting errors. ICI exposure was categorized as: anti-PD-1 (pembrolizumab, nivolumab, cemiplimab, and dostarlimab), anti-PD-L1 (atezolizumab, durvalumab, and avelumab), anti-CTLA-4 (ipilimumab and tremelimumab), or combination therapy.^[13]^ Vitiligo cases were identified using comprehensive search strategies encompassing MedDRA preferred terms: “vitiligo,” “leukoderma,” “skin hypopigmentation,” “skin depigmentation,” and ocular pigmentary changes (“retinal hypopigmentation,” “iris hypopigmentation,” “eyelid hypopigmentation,” and “eyelash hypopigmentation”).^[14,15]^ To minimize bias introduced by different reporters, we corrected for indications and medications taken as indicated in the acquired reports. Case reports of ICI-induced vitiligo adverse reactions (N = 591) were retrieved for further analysis.

### 2.3. Disproportionality analysis

We employed 4 established signal detection algorithms to evaluate disproportionate reporting of vitiligo across ICI treatment strategies: reporting odds ratio (ROR), proportional reporting ratio, Information Component (IC), and empirical Bayes geometric mean. A positive safety signal required satisfaction of all 4 algorithmic threshold criteria (Table 2).^[16]^ For TCGA integration analyses, we calculated ROR exclusively as the primary effect measure given its established performance in pharmacovigilance studies.^[17]^ Descriptive analyses encompassed demographic characteristics (gender, age categorized as 18–64, 65–79, and 80–100 years), geographic region (Americas, Europe, Asia, Oceania, Africa, and other), report receipt year, ICI treatment strategy, clinical outcomes (death, hospitalization, life-threatening, and other), and indication organ system.

### 2.4. Pan-cancer transcriptomic analysis

Normalized enrichment scores for 1387 canonical pathways across 33 cancer types were downloaded from the UCSC Xena Pan-Cancer Atlas Hub (https://xenabrowser.net). Sample identifiers were mapped to TCGA project abbreviations, and median single-sample gene set enrichment analysis scores were calculated for each pathway-cancer type combination using R software (version 4.5.2; R Core Team, Vienna, Austria). Analyses were restricted to 7 cancer types with substantial ICI utilization and available transcriptomic data: breast invasive carcinoma, uterine corpus endometrial carcinoma, liver hepatocellular carcinoma, lung adenocarcinoma, lung squamous cell carcinoma, skin cutaneous melanoma, and kidney renal clear cell carcinoma. The association between ICI-induced vitiligo ROR and pathway activation levels was evaluated using Spearman rank correlation coefficient, with statistical significance defined as 2-tailed *P* < .05.

### 2.5. Statistical analysis

Categorical variables were compared using chi-square or Fisher exact tests as appropriate. TTO was defined as the interval between ICI initiation and vitiligo onset, estimated using the Kaplan–Meier method and compared across groups using the Kruskal–Wallis test with Dunn post hoc correction for multiple comparisons. Correlation analyses employed Spearman rank test to accommodate nonnormal distributions. All statistical analyses and visualizations were performed using R (version 4.5.2; https://www.r-project.org/) and GraphPad Prism (version 8.0; GraphPad Software, La Jolla).

## 3. Results

### 3.1. Descriptive analysis of ICI-induced vitiligo cases

Between January 2015 and September 2025, 591 cases of ICI-induced vitiligo were identified in the FAERS database. Demographic and clinical characteristics are summarized in Table 1. Male patients accounted for 291 cases (49.2%), while female patients contributed 231 cases (39.1%). Age stratification revealed that patients aged 18 to 64 years represented the largest proportion (245/591, 41.5%) and exhibited the highest crude incidence rate (0.33%). Geographic distribution analysis demonstrated that European reports predominated (215/591, 36.4%), followed by North America; however, South America exhibited the highest regional incidence rate (0.4%). Temporal analysis indicated a declining trend in vitiligo reporting from 2015 to 2025, despite increasing ICI utilization, potentially reflecting improved recognition, evolving reporting practices, or enhanced patient selection.^[18]^ Clinical outcomes were notable: death occurred in 4.7% (28/591) of cases, while 12.5% (74/591) required hospitalization, underscoring that vitiligo may herald or coincide with severe systemic irAEs.^[19]^

**Table 1:** Clinical characteristics of patients treated with ICIs collected from the FAERS database (January 2015–September 2025).

Clinical characteristics	Vitiligo	Nonvitiligo	Incidence of vitiligo	*P* value
Gender				
Male	291 (49.2%)	119,476 (51.4%)	0.24%	.55
Female	231 (39.1%)	86,083 (37%)	0.27%	
Unknown	69 (11.7%)	26,919 (11.6%)	0.26%	
Age				
18–64	245 (41.5%)	74,452 (32%)	0.33%	<.0001
65–79	179 (30.3%)	77,584 (33.4%)	0.23%	
80–100	32 (5.4%)	13,403 (5.8%)	0.24%	
Unknown	135 (22.8%)	67,039 (28.8%)	0.20%	
Region				
Europe	215 (36.4%)	63,698 (27.4%)	0.34%	<.0001
North America	182 (30.8%)	80,399 (34.6%)	0.23%	
South America	13 (2.2%)	3254 (1.4%)	0.40%	
Asia	133 (22.5%)	57,605 (24.8%)	0.23%	
Oceania	11 (1.9%)	3802 (1.6%)	0.29%	
Africa	1 (0.2%)	397 (0.2%)	0.25%	
Unknown	36 (6.1%)	23,323 (10%)	0.15%	
Received year				
2015	33 (11.1%)	3590 (2.7%)	0.91%	<.0001
2016	38 (12.8%)	7488 (5.6%)	0.50%	
2017	48 (16.2%)	10,822 (8.1%)	0.44%	
2018	45 (15.2%)	12,426 (9.3%)	0.36%	
2019	40 (13.5%)	14,436 (10.8%)	0.28%	
2020	17 (5.7%)	13,680 (10.2%)	0.12%	
2021	18 (6.1%)	16,182 (12.1%)	0.11%	
2022	23 (7.7%)	18,465 (13.8%)	0.12%	
2023	23 (7.7%)	17,189 (12.8%)	0.13%	
2024	11 (3.7%)	13,533 (10.1%)	0.08%	
2025	1 (0.3%)	6340 (4.7%)	0.02%	
Outcome				
Hospitalization	74 (12.5%)	67,973 (29.2%)	0.11%	<.0001
Death	28 (4.7%)	33,584 (14.4%)	0.08%	
Life-threatening	9 (1.5%)	5406 (2.3%)	0.17%	
Other	398 (67.3%)	100,050 (43%)	0.40%	
Unknown	82 (13.9%)	25,465 (10.9%)	0.32%	
ICI treatment strategy				
Anti-PD-1	358 (60.6%)	147,687 (63.5%)	0.24%	<.0001
Anti-PD-L1	29 (4.9%)	46,564 (20%)	0.06%	
Anti-CTLA-4	27 (4.6%)	7179 (3.1%)	0.37%	
Combination therapy	177 (29.9%)	31,048 (13.4%)	0.57%	
Indication organ				
Skin	383 (64.8%)	26,306 (11.3%)	1.44%	<.0001
Lung	53 (9%)	60,406 (26%)	0.09%	
Mammary gland	15 (2.5%)	10,024 (4.3%)	0.15%	
Liver	15 (2.5%)	11,731 (5%)	0.13%	
Kidney	11 (1.9%)	22,615 (9.7%)	0.05%	
Stomach	6 (1%)	9244 (4%)	0.06%	
Head and neck	5 (0.8%)	4411 (1.9%)	0.11%	
Bladder	5 (0.8%)	3660 (1.6%)	0.14%	
Lymph node	2 (0.3%)	3408 (1.5%)	0.06%	
Prostate	1 (0.2%)	1592 (0.7%)	0.06%	
Blood	1 (0.2%)	944 (0.4%)	0.11%	
Ovary	0	2107 (0.9%)	0	
Brain	0	1104 (0.5%)	0	
Colorectal	0	3223 (1.4%)	0	
Pancreas	0	244 (0.1%)	0	
Unspecified	78 (13.2%)	60,629 (26.1%)	0.13%	
Unknown	16 (2.7%)	10,830 (4.7%)	0.15%	

**Figure 2.**
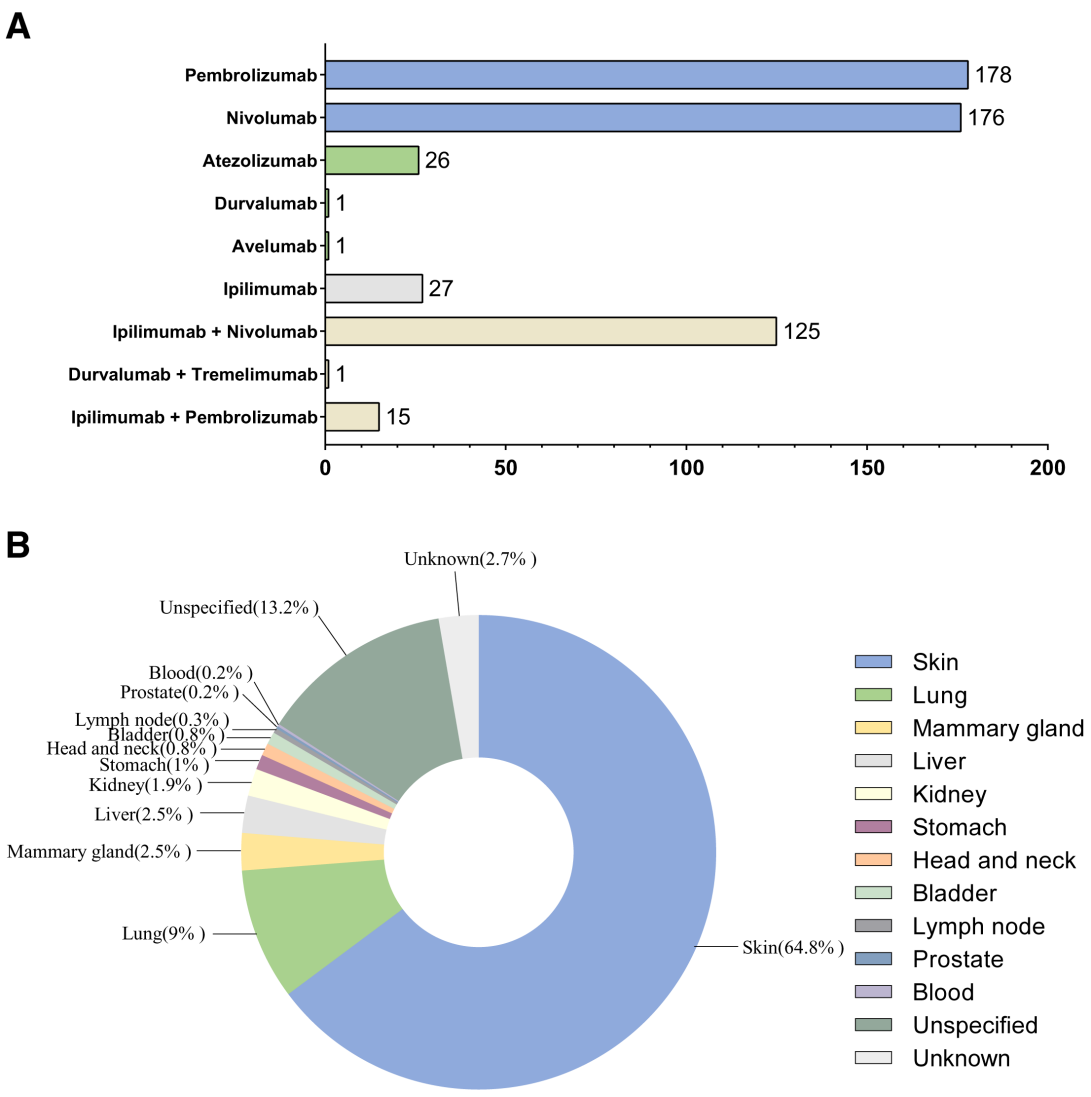
Distribution of ICI-induced vitiligo. (A) Bar chart shows the number of ICI-induced vitiligo reports for main ICI treatment strategies. (B) Pie chart shows the proportional composition of patient’s cancer original sites. ICI = immune checkpoint inhibitor.

Analysis of treatment strategies revealed that combination therapy exhibited the highest vitiligo incidence (0.57%), while anti-PD-L1 monotherapy demonstrated the lowest (0.06%). AntiPD-1 agents accounted for the largest absolute number of cases (358/591, 60.6%), reflecting their widespread clinical use, followed by combination therapy (177/591, 29.9%), anti-PD-L1 (29/591, 4.9%), and anti-CTLA-4 monotherapy (27/591, 4.6%) (Fig. 2A). Regarding underlying malignancies, skin cancers (predominantly melanoma) represented 64.8% (383/591) of cases, followed by lung malignancies at 9.0% (53/591) (Fig. 2B), consistent with established ICI indications and the melanocytic origin of cutaneous malignancies.^[20]^

### 3.2. Disproportionality analysis of ICI treatment strategies

Disproportionality analysis employing 4 algorithmic criteria demonstrated robust safety signals for vitiligo across ICI classes (Table 2). Total ICI usage and 3 major therapeutic categories – anti-PD-1, anti-CTLA-4, and combination therapy – satisfied all 4 threshold criteria for positive signal detection.^[21]^ Among specific regimens, ipilimumab combined with pembrolizumab exhibited the highest ROR (97.2, 95% CI: 58.1–162.4), indicating substantially disproportionate vitiligo reporting relative to other adverse events (Table 3). In monotherapy analyses, ipilimumab demonstrated the highest ROR (13.7, 95% CI: 9.4–20.0), followed by nivolumab (11.1, 95% CI: 9.5–12.9), pembrolizumab (7.8, 95% CI: 6.7–9.1), and atezolizumab (2.9, 95% CI: 2.0–4.3). Agents with limited vitiligo case numbers (cemiplimab, dostarlimab, durvalumab, avelumab, and tremelimumab) did not meet signal threshold criteria, likely reflecting their more recent approval and lower cumulative exposure.^[22]^ These results indicate that ICI combination therapy and sequential use of similar agents confer elevated vitiligo risk, supporting the hypothesis that synergistic or cumulative immune checkpoint blockade enhances autoimmune targeting of melanocytes.

**Table 2:** Summary of algorithms used for signal detection.

Algorithms	Equation	Threshold value
ROR	ROR = *ad*/*bc*	N ≥ 2, 95% CI > 1
	95% CI = e^ln(ROR)±^1^.96(1/*a*+1/*b*+1/*c*+1/*d*)0.5^	
PRR	PRR = *a*(*c* + *d*)/*c*/(*a* + *b*)	N ≥ 2, PRR ≥ 2, χ^2^ ≥ 4
	χ^2^ = [(*ad* − *bc*)^2^](*a* + *b* + *c* + *d*)/[(*a* + *b*)(*c* + *d*)(*a* + *c*)(*b* + *d*)]	
BCPNN	IC = log_2_*a*(*a* + *b* + *c* + *d*)(*a* + *c*)(*a* + *b*)	IC025 > 0
	IC025 = e^(ln (IC)−^1^.96(1/*a*+1/*b*+1/*c*+1/*d*)0.5^^)^	
MGPS	EBGM = *a*(*a* + *b* + *c* + *d*)/(*a* + *c*)/(*a* + *b*)	EBGM05 > 2
	EBGM05 = e^(ln (EBGM)−^1^.64(1/*a*+1/*b*+1/*c*+1/*d*)0.5^^)^	

BCPNN = Bayesian confidence propagation neural network, CI = confidence interval, EBGM = empirical Bayes geometric mean, IC = information component, MGPS = multi-item gamma Poisson shrinker algorithms, ROR = reporting odds ratio, PRR = proportional reporting ratio.

**Table 3:** Disproportion analysis of different ICI treatment strategies and vitiligo.

ICIs treatment strategy	Number of cases	Events of vitiligo	ROR (95% CI)	PRR (χ^2^)	IC (IC025)	EBGM (EBGM05)
Total	233,069	591	9.3 (8.6–10.1)	9.3 (4223.5)	3.2 (3.1)	9.0 (8.3)
Anti-PD-1	148,045	358	9.4 (8.4–10.4)	9.3 (2425.4)	3.1 (3.0)	8.6 (7.7)
Pembrolizumab	84,597	178	7.8 (6.7–9.1)	7.8 (1003.9)	2.9 (2.7)	7.5 (6.4)
Nivolumab	58,940	176	11.1 (9.5–12.9)	11.0 (1537.4)	3.4 (3.2)	10.6 (9.1)
Cemiplimab	2567	0	–	–	–	–
Dostarlimab	1494	0	–	–	–	–
Sequential use of PD-1 inhibitors	447	4	32.1 (12.0–85.9)	31.8 (119.2)	5.0 (3.7)	31.8 (11.9)
Anti-PD-L1	46,593	29	2.2 (1.5–3.2)	2.2 (19.3)	1.1 (0.6)	2.2 (1.5)
Atezolizumab	31,495	26	2.9 (2.0–4.3)	2.9 (33.2)	1.6 (1.0)	2.9 (2.0)
Durvalumab	11,543	1	0.3 (0.043–2.2)	0.3 (1.6)	−1.7 (−3.7)	0.3 (0.043)
Avelumab	3473	1	1.0 (0.1–7.3)	1.0 (0.0005)	0.032 (−2.0)	1.0 (0.1)
Sequential use of PD-L1 inhibitors	82	1	43.8 (6.13–15.0)	43.3 (41.3)	5.4 (3.4)	43.3 (6.0)
Anti-CTLA-4	7206	27	13.4 (9.2–19.6)	13.4 (307.5)	3.7 (3.2)	13.3 (9.1)
Ipilimumab	7075	27	13.7 (9.4–20.0)	13.6 (314.1)	3.8 (3.2)	13.5 (9.3)
Tremelimumab	130	0	–	–	–	–
Sequential use of CTLA-4 inhibitors	1	0	–	–	–	–
Combination therapy	31,225	177	21.1 (18.2–24.6)	21.0 (3225.1)	4.3 (4.1)	20.1 (17.3)
Ipilimumab + nivolumab	26,147	125	17.6 (14.7–21.0)	17.5 (1883.1)	4.1 (3.8)	17.0 (14.2)
Durvalumab + tremelimumab	2884	1	1.2 (0.2–8.7)	1.2 (0.043)	0.3 (−1.7)	1.2 (0.2)
Ipilimumab + pembrolizumab	565	15	97.2 (58.1–162.4)	94.6 (1384.5)	6.6 (5.8)	94.3 (56.4)

CI = confidence interval, CTLA-4 = cytotoxic T-lymphocyte-associated protein 4, EBGM = empirical Bayes geometric mean, IC = information component, ICI = immune checkpoint inhibitor, PD-1 = programmed cell death protein 1, PD-L1 = programmed death-ligand 1, ROR = reporting odds ratio, PRR = proportional reporting ratio.

### 3.3. TTO analysis of ICI-induced vitiligo

**Figure 3.**
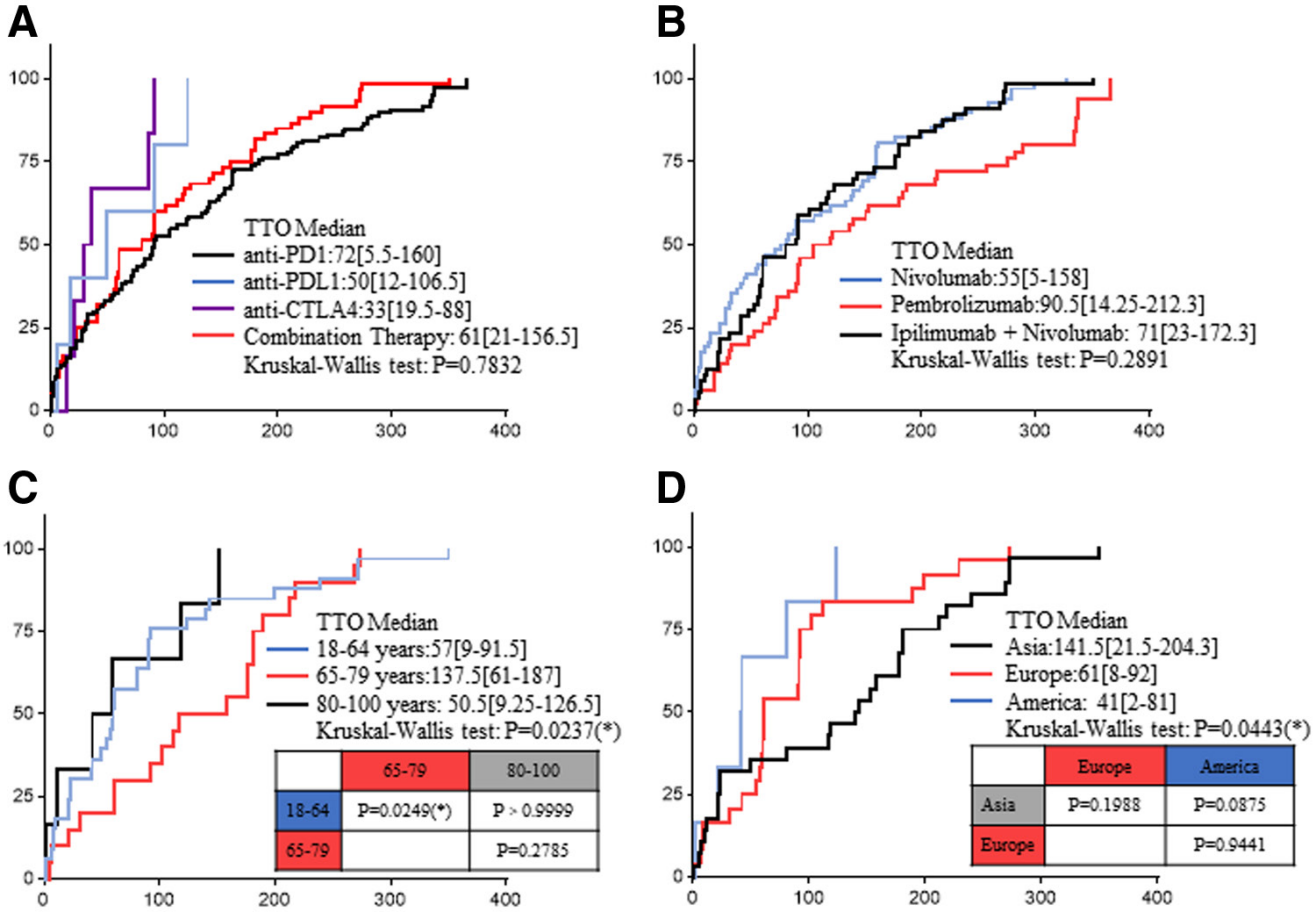
Time-to-onset of ICI-induced vitiligo. (A) TTO analysis of ICI classes. (B) TTO analysis of 3 most frequent regimens. (C) TTO analysis of age. (D) TTO analysis of geographical region. CTLA-4 = cytotoxic T-lymphocyte-associated protein 4, ICI = immune checkpoint inhibitor, PD-1 = programmed cell death protein 1, PD-L1 = programmed death-ligand 1, TTO = time-to-onset.

Median TTO varied significantly across ICI classes: anti-CTLA-4 monotherapy demonstrated the shortest median TTO (33 days, interquartile range [IQR] not specified), followed by anti-PD-L1 (50 days), combination therapy (61 days), and anti-PD-1 (72 days) (Fig. 3A). Analysis of the 3 most frequent regimens revealed nuanced temporal patterns. Nivolumab monotherapy exhibited the shortest median TTO (55 days, IQR 5–158), compared to pembrolizumab (90 days, IQR 14.25–212.3) and ipilimumab–nivolumab combination (71 days, IQR 23–172.3), although between-group differences did not achieve statistical significance by Kruskal–Wallis test (Fig. 3B).

Age-stratified analysis demonstrated significant heterogeneity: patients aged 18 to 64 years exhibited a median TTO of 57 days (IQR 9–91.5), compared to 137.5 days (IQR 61–187) in the 65 to 79 years group and 50.5 days (IQR 9.25–126.5) in the 80 to 100 years group. Notably, a significant difference was observed between the 18 to 64 and 65 to 79 years groups (*P* = .0249), suggesting immunosenescence-related delays in autoimmune manifestation (Fig. 3C). Geographic variation was also significant: patients from the Americas demonstrated the shortest median TTO (41 days, IQR 2–81), followed by Europe (61 days, IQR 8–92) and Asia (141.5 days, IQR 21.5–204.3; Kruskal–Wallis *P* = .0443) (Fig. 3D). These findings implicate genetic, environmental, or healthcare system factors in modulating irAE kinetics.

### 3.4. Biological mechanisms underlying ICI-induced vitiligo

**Figure 4.**
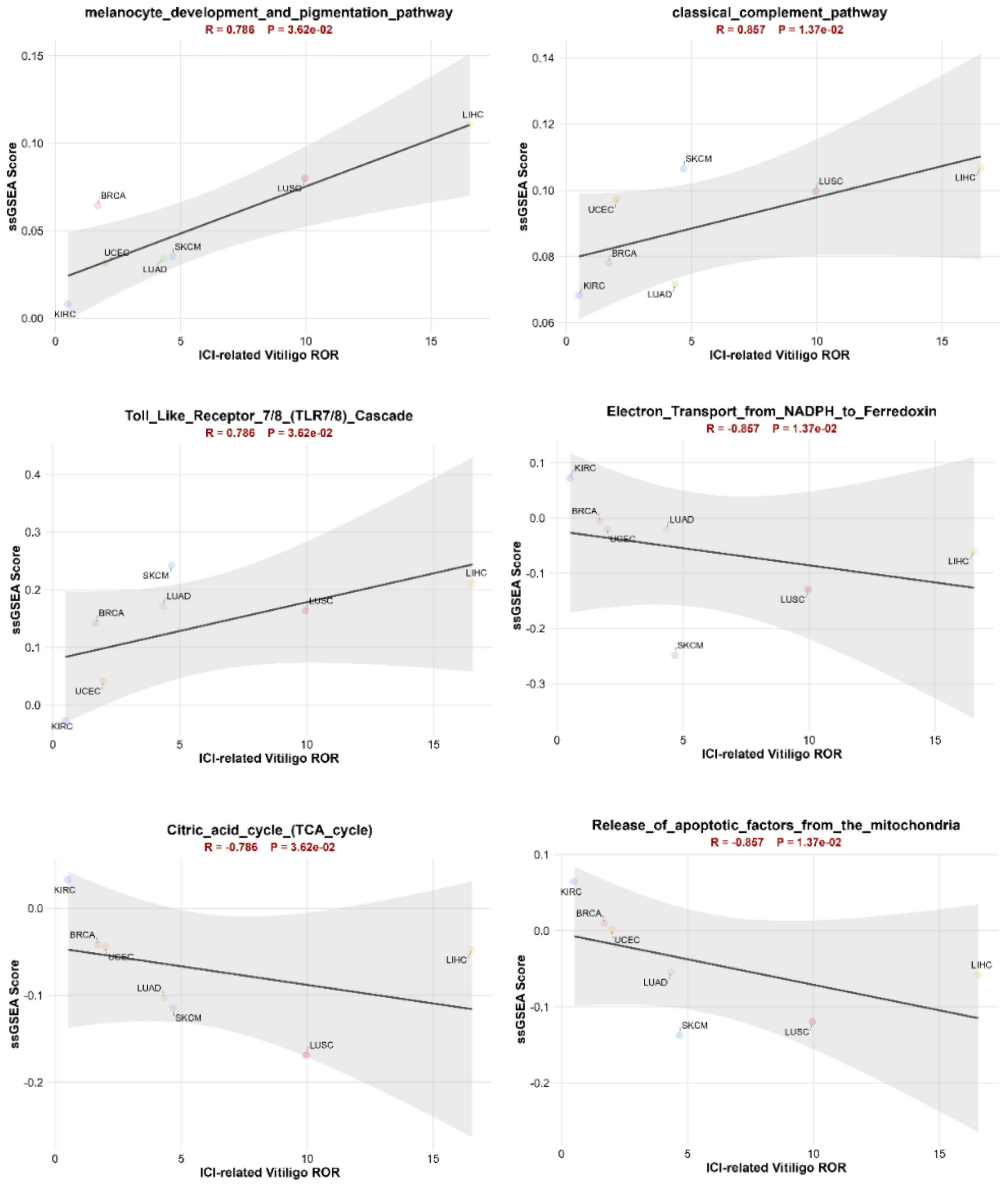
Correlation between ICI-induced vitiligo and pan-cancer biological features. ICI = immune checkpoint inhibitor, NADPH = nicotinamide adenine dinucleotide phosphate, ROR = reporting odds ratio, ssGSEA = single-sample gene set enrichment analysis, TCA = tricarboxylic acid.

Pan-cancer analysis revealed significant associations between vitiligo reporting rates and specific biological pathways (Fig. 4). The ROR for ICI-induced vitiligo exhibited a strong positive correlation with melanocyte development and pigmentation pathway activation, supporting the antigenic mimicry hypothesis wherein tumors with high melanocytic differentiation prime cross-reactive immune responses. Additionally, significant positive correlations were observed with the classical complement pathway and toll-like receptor 7/8 (TLR7/8) signaling cascade, implicating innate immune activation in vitiligo pathogenesis. Complement-mediated cytotoxicity and TLR-driven dendritic cell maturation may amplify autoimmune targeting of melanocytes. Conversely, significant negative correlations were identified with mitochondrial electron transport from nicotinamide adenine dinucleotide phosphate to ferredoxin, tricarboxylic acid cycle, and release of apoptotic factors from mitochondria, suggesting that reduced baseline mitochondrial stress or altered apoptotic signaling may paradoxically increase susceptibility to immune-mediated melanocyte destruction, potentially through modified antigen presentation or danger signal release.

## 4. Discussion

This comprehensive pharmacovigilance analysis of the FAERS database identified 591 cases of ICI-induced vitiligo reported over a decade, providing novel insights into the clinical epidemiology, temporal dynamics, and biological mechanisms of this distinctive irAE. By integrating real-world safety data with pan-cancer transcriptomic profiles, we demonstrate that vitiligo risk varies substantially across ICI treatment strategies, exhibits demographic and geographic heterogeneity in onset kinetics, and correlates with specific melanocyte-related and innate immune pathways.

Our findings confirm and extend previous observations regarding ICI-induced vitiligo. In a recent pharmacovigilance study spanning 2015 to 2024, Tang et al reported that ICI-treated patients who developed vitiligo exhibited significantly lower mortality compared with those without vitiligo (8.82% vs 26.4%), advancing the hypothesis that vitiligo may serve as a cross-tumor prognostic biomarker for immunotherapy efficacy. Nevertheless, their analysis was restricted to ICI monotherapy, and the underlying biological mechanisms remained unexplored.^[23]^ In our study, disproportionality analysis reveals that anti-CTLA-4 and combination therapies confer substantially higher relative risks. The exceptional ROR observed for ipilimumab–pembrolizumab combination (97.2) aligns with clinical trial evidence of synergistic toxicity with dual checkpoint blockade, likely resulting from convergent disruption of peripheral tolerance mechanisms.^[24,25]^ The significant proportion of cases arising from nonmelanoma cancers (35.2%) expands the clinical spectrum of ICI-induced vitiligo beyond its well-characterized association with melanoma. This observation suggests that melanocyte-directed autoimmunity can be primed by nonmelanocytic tumors, possibly through neoantigen mimicry or bystander activation mechanisms. The declining incidence trend from 2015 to 2025 may indicate improved patient selection, enhanced dermatologic monitoring, or reporting artifacts, warranting continued surveillance.^[19]^ Notably, our finding that 12.5% of vitiligo cases required hospitalization and 4.7% were associated with fatal outcomes challenges the perception of vitiligo as a purely cosmetic irAE. While these severe outcomes likely reflect concurrent systemic irAEs rather than vitiligo per se, they underscore the necessity for comprehensive evaluation when vitiligo is identified.

The marked variation in TTO across ICI classes carries important implications for clinical monitoring. The rapid onset associated with anti-CTLA-4 (median 33 days) aligns with its mechanism of action in lymphoid tissues during T-cell priming, whereas the delayed onset with anti-PD-1 (median 72 days) reflects peripheral tissue modulation of previously activated T cells. These pharmacodynamic distinctions should inform the timing and intensity of dermatologic surveillance protocols. The significant age-related delay in TTO among patients aged 65 to 79 years (137.5 days vs 57 days in younger adults) suggests immunosenescence-associated alterations in T-cell kinetics, including reduced thymic output and narrowed T-cell receptor diversity.^[26,27]^ Conversely, the shortest TTO observed in octogenarians (50.5 days) may reflect selection bias for robust responders or distinct immune aging trajectories. Geographic variation in TTO, with Asian patients demonstrating substantially delayed onset, implicates genetic factors – such as human leukocyte antigen polymorphisms – or environmental modifiers in irAE pathogenesis.^[28,29]^

Integration of pharmacovigilance and transcriptomic data provides novel mechanistic insights into ICI-induced vitiligo. The strong correlation with melanocyte development pathways validates the central role of melanocyte-lineage antigens in driving cross-reactive autoimmunity, consistent with the observed association between vitiligo and antitumor response in melanoma.^[30,31]^ The implicated complement and TLR7/8 pathways suggest that innate immune activation serves as a critical amplifier of melanocyte-directed autoimmunity.^[32,33]^ Complement-mediated opsonization and lysis, coupled with TLR-driven type I interferon responses, may lower the threshold for T-cell-mediated melanocyte destruction.^[34]^ These findings identify potential therapeutic targets for vitiligo prevention or treatment, including complement inhibitors or TLR antagonists, although careful consideration would be required to avoid compromising antitumor immunity. The unexpected negative correlations with mitochondrial pathways warrant further investigation. Reduced mitochondrial stress responses might paradoxically enhance immunogenicity through altered reactive oxygen species signaling or modified antigen processing, suggesting a complex interplay between cellular metabolism and immune recognition.^[35]^

Several methodological limitations should be acknowledged. First, FAERS is a passive surveillance system subject to underreporting, reporting bias, and incomplete data capture, which may affect incidence estimates and disproportionality measures. The absence of a defined denominator precludes calculation of absolute incidence rates; ROR values reflect relative rather than absolute risk. Second, causality cannot be definitively established due to lack of detailed clinical information, including histopathological confirmation of vitiligo, prior autoimmune history, concomitant medications, and underlying disease status. The inability to distinguish segmental from nonsegmental vitiligo or assess disease severity limits clinical granularity. Third, the pan-cancer transcriptomic analysis relies on pretreatment tumor samples and aggregated pathway scores, which may not reflect dynamic changes during ICI therapy or capture intratumor heterogeneity. The restricted analysis to 7 cancer types limits generalizability. Fourth, temporal and geographic variations may reflect differential reporting practices, healthcare access, or regulatory environments rather than true biological differences. The observed decline in reporting could represent reporting fatigue or shifting clinical attention toward more severe irAEs.

This large-scale pharmacovigilance study provides a comprehensive characterization of ICI-induced vitiligo, demonstrating significant heterogeneity in risk across treatment strategies, distinct temporal patterns influenced by demographic and geographic factors, and mechanistic links to melanocyte-specific and innate immune pathways. These findings enhance understanding of immunotherapy-induced autoimmunity, inform risk-stratified monitoring approaches, and identify potential targets for preventive and therapeutic intervention. As ICIs continue to expand across oncology indications, recognizing and managing vitiligo as a clinically significant irAE remains essential for optimizing patient outcomes in the era of cancer immunotherapy.

## 5. Conclusion

This study performed a pharmacovigilance analysis based on real-world adverse event reports from FAERS to explore vitiligo associated with immune checkpoint inhibitors. The incidence rate and onset time of vitiligo caused by different ICIs vary, and there are also differences among patients of different ages and geographical regions. The underlying mechanism may be related to biological processes such as melanocyte development and innate immune activation. These findings supplement evidence from clinical studies that often involve limited sample sizes and observation periods. The results provide valuable insights for improving clinical monitoring and promoting safer use of immune checkpoint inhibitors. Continuous evaluation of the safety and efficacy of the drug remains essential.

## Acknowledgments

We are grateful to the FAERS database for enabling the data analysis. This study was supported by Medical Science and Technology Foundation of Guangdong Province (No. A2021491) and Guangdong Provincial Innovative Talent Cultivation Overseas Distinguished Teacher Project (No. 109226955038).

## Author contributions

**Data curation:** Jingchang Ma, Ya Luo.

**Formal analysis:** Jingchang Ma, Xiaojun Li.

**Methodology:** Jingchang Ma, Jinyi Li, Mingjun Hou.

**Software:** Xiaojun Li.

**Writing – original draft:** Jingchang Ma, Zhibo Cai.

**Writing – review & editing:** Dingheng Zhu.
